# Context matters! sources of variability in weekend physical activity among families: a repeated measures study

**DOI:** 10.1186/s12889-017-4232-9

**Published:** 2017-04-18

**Authors:** Robert J. Noonan, Stuart J. Fairclough, Zoe R. Knowles, Lynne M. Boddy

**Affiliations:** 1 Physical Activity Exchange, Research Institute for Sport and Exercise Sciences, Liverpool John Moores University, Liverpool, UK; 2 Department of Sport and Physical Activity, Edge Hill University, Ormskirk, UK; 3 Department of Physical Education and Sport Sciences, University of Limerick, Limerick, Ireland

**Keywords:** Physical activity, Children, Family, Accelerometer, Diary, Raw, Context, ActiGraph

## Abstract

**Background:**

Family involvement is an essential component of effective physical activity (PA) interventions in children. However, little is known about the PA levels and characteristics of PA among families. This study used a repeated measures design and multiple data sources to explore the variability and characteristics of weekend PA among families.

**Methods:**

Families (including a ‘target’ child aged 9–11 years, their primary caregiver(s) and siblings aged 6–8 years) were recruited through primary schools in Liverpool, UK. Participants completed a paper-based PA diary and wore an ActiGraph GT9X accelerometer on their left wrist for up to 16 weekend days. ActiGraph.csv files were analysed using the R-package GGIR version 1.1–4. Mean minutes of moderate-to-vigorous PA (MVPA) for each weekend of measurement were calculated using linear mixed models, and variance components were estimated for participant (inter-individual), weekend of measurement, and residual error (intra-individual). Intraclass correlation coefficients (ICC) were calculated from the proportion of total variance accounted for by inter-individual sources, and used as a measure of reliability. Diary responses were summed to produce frequency counts. To offer contextual insight into weekend PA among family units, demographic, accelerometer, and diary data were combined to form two case studies representative of low and high active families.

**Results:**

Twenty-five participants from 7 families participated, including 7 ‘target’ children (mean age 9.3 ± 1.1 years, 4 boys), 6 siblings (mean age 7.2 ± 0.7 years; 4 boys) and 12 adults (7 mothers and 5 fathers). There was a high degree of variability in target children’s (ICC = 0.55), siblings (ICC = 0.38), and mothers’ MVPA (ICC = 0.58), but not in fathers’ MVPA (ICC = 0.83). Children’s weekend PA was mostly unstructured in nature and undertaken with friends, whereas a greater proportion of parents’ weekend PA was undertaken alone in structured settings. The family case studies demonstrated that in the selected cases MVPA levels and variability across weekends were contingent on mode of PA participation.

**Conclusions:**

These novel findings enhance understanding of the variability and characteristics of weekend PA among family units. The study demonstrates the utility of PA diaries in conjunction with accelerometers to provide understanding of the mode and contexts of out-of-school and family-based PA.

## Background

Childhood is an important developmental stage during which health and lifestyle behaviours such as physical activity (PA) are established [1, 2]. Regular PA during childhood provides broad ranging health benefits [3, 4]. To achieve and maintain these benefits, the UK Chief Medical Officers recommend that children accumulate at least 1 h of moderate-to-vigorous PA (MVPA) each day, and minimise time spent in sedentary behaviours [5]. However, there is strong evidence to suggest that few children in the UK [6, 7] and other developed countries including America [8], Australia [9] and Canada [10] currently achieve the recommended levels of PA to benefit their health.

Children’s PA comprises a broad range of structured (e.g., organised sport) and unstructured activities (e.g., active travel, outdoor play) that take place in a variety of settings [11, 12]. The school setting provides a range of PA opportunities for children and contributes a significant proportion of their daily PA [13, 14]. These school-based PA opportunities are inclusive to all, as they form part of the school curriculum (e.g., physical education), discretionary time in school (e.g., recess play), and after-school provision (e.g., organised after-school activities) during the school week. In contrast, opportunities for PA on weekend days are strongly influenced by parental encouragement (e.g., positive verbal reinforcement) and support (e.g., payment of club subscriptions, transport to and from provision) [15, 16], as well as constraints on individual choice (e.g., access to garden/backyard) [17–19]. Given that children also experience less structure and routine, and thus more behavioural choice on weekend days compared to weekdays, it is likely that their PA levels will vary considerably from weekend to weekend [20, 21]. However, most previous studies have been limited to measuring PA once over a 7-day period encompassing weekdays and weekend days [22–28]. Consequently, how representative this one-off measurement of weekend PA is of typical weekend PA behaviour remains unknown. Thus, further research is needed to specifically examine the variability of weekend PA from repeated measurements.

The weekend is an important time period for PA promotion. Firstly, children tend to accumulate the least amount of daily MVPA on weekend days [29, 30]. Secondly, during the school term, weekends offer children the most discretionary time for leisure activity, and opportunities for the whole family to be physically active can be implemented more easily on weekends [31]. Family involvement is an essential component of effective PA interventions in children [32–34]. Family-based PA interventions that encourage PA co-participation between children and parents [35, 36] and among siblings [37] may yield beneficial effects as both are associated with higher child PA. Understanding the PA patterns of families is necessary for designing effective family-based PA interventions. However, little is known about the PA behaviours and habitual routines of families on weekends. To date, family-focused PA research has been qualitative in nature [31, 33, 38–42]. None of these studies have involved all household family members as participants, or included objective assessments of PA.

To date, most family-focused PA interventions have followed ‘fixed prescriptions’ and in doing so have not engaged with families prior to intervention delivery (e.g., [43–45]). Intervention programmes that are tailored to individual family needs and characteristics may help to overcome key intervention challenges including recruitment and engagement, and thus could improve intervention efficacy [33, 46]. Moreover, research in this area is often based on group-level comparisons drawn from “one-off” assessments of PA which may not present a true reflection of a child’s or parent’s habitual level of PA. The inclusion of whole families comprising target children, parents, and siblings in the same study offers an original way in which to explore the characteristics of family unit weekend PA, which may help inform family-focused PA intervention design. This study, therefore, assessed ‘target’ children’s PA, and their siblings’ and parents’ PA over eight weekends using accelerometry and PA diaries. The aims of the study were twofold: 1. To investigate the stability of weekend MVPA among target children, siblings, and parents using repeated measures raw accelerometer data, and 2. To offer contextual insight into the characteristics of weekend PA amongst one representative low active family and one high active family.

## Methods

### Participants

Families including a ‘target’ child aged 9–11 years, their primary caregiver(s) (herein referred to as parents) and siblings aged 6–8 years were recruited through primary schools in Liverpool, UK. Three primary schools located in areas representing varying socioeconomic status based on the UK Indices of Multiple Deprivation (IMD) (SES; IMD = 12.0 (UK tertile 2), IMD = 38.4 (UK tertile 5), and IMD = 43.6 (UK tertile 5)) were approached as convenience samples and agreed to participate in the study. The selected schools had participated in previous research studies led by the first author. Information flyers, written study information and a questionnaire were issued to all Year 5 and 6 children (*n* = 210) in participating schools to take home for their parent to complete and return upon completion. All school aged siblings (>4 years and <18 years) and parents living in the same household were invited to take part. Minimum inclusion criteria for a family required one child participant aged 9–11 years and at least one parent participant. Completed informed parental consent and child assent were returned from seven families. The first author contacted consenting parents via SMS to arrange a suitable time to visit all family members at their home address. The study received institutional ethics approval (reference number: 15/SPS/023) and data collection took place between June 2015 and April 2016. Each family received a £50 high street shopping voucher after data collection in return for their participation.

### Measures

#### Socioeconomic status

Parent reported home postcodes were imported into the GeoConvert application [47] to calculate area level SES based on the 2015 IMD. The IMD is a UK Government produced measure comprising seven areas of deprivation (income, employment, health, education, housing, environment, and crime). Higher SES was represented by lower deprivation scores. Individual level SES was assessed using the highest level of education for each family. Responses included; high school, college, university, higher degree [48].

#### Anthropometrics

Anthropometric assessments were taken at home addresses for all participants by the first author using standard procedures [49]. Participant sex and age were also recorded. Child stature, sitting height and body mass were measured using a portable stadiometer (Leicester Height Measure, Seca, Birmingham, UK) and an electronically calibrated digital scale (Tanita WB-110A, Tanita Europe, The Netherlands) to the nearest 0.1 cm and 0.1 kg, respectively. Child leg length was calculated by subtracting sitting height from stature. Body mass index (BMI) was calculated from height and weight (kg/m^2^) and BMI z-scores were assigned to each child [50]. Age and sex specific BMI cut-points were then used to classify child weight status [51]. Gender-specific regression equations [52] were used to calculate somatic maturity (years from peak height velocity). Waist circumference was measured to the nearest 0.1 cm using a non-elastic measuring tape (Seca, Birmingham, UK). Parent stature and mass were measured using the same procedures. BMI was calculated from height and weight (kg/m^2^) and BMI cut-points were used to classify parent weight status [53].

#### Habitual physical activity

PA was assessed using the ActiGraph GT9X accelerometer which features ActiGraph’s validated tri-axial accelerometer and data filtering technology (GT9X, theActiGraph.com, FL, USA) [54, 55]. The GT9X model was selected because it measures raw accelerations and is worn on the wrist which is associated with improved device wear [56]. Participants wore the accelerometer on their left wrist during waking hours for two weekend days. They were instructed to only remove the monitor during water-based activities and when sleeping. Verbal and written instructions for care and placement of the monitor were given to participants. After the two measurement days accelerometers were collected from home addresses, the data downloaded, and then returned to participants on the subsequent Friday to wear again on weekend days. This process was repeated on four consecutive occasions in one season and on a further four consecutive occasions in the subsequent season, resulting in a total of 16 weekend measurement days per participant. Four families completed measures throughout June/July (summer) and November/December (autumn/winter) 2015 and three families completed measures throughout October/November (autumn) 2015 and March/April (spring) 2016. The accelerometers were set to record data at a frequency of 30 Hz, and were marked with separate color-coded stickers for parents and children to avoid any mistaken cross usage. Data collection took place during the regular school term so activities were representative of usual free-living activities.

ActiGraph data were downloaded using ActiLife v. 6.11.4 (ActiGraph, Pensacola, FL), saved in raw format as GT3X files, and subsequently converted to CSV format to facilitate raw data processing. Raw data files were processed in R (http://cran.r-project.org) using the GGIR package (version 1.2–0) which converted raw triaxial acceleration values into one omnidirectional measure of acceleration, termed the signal vector magnitude (SVM). SVM was calculated from raw accelerations from the three axes minus 1 *g* which represents the value of gravity (i.e., SVM = √(x^2^ + y^2^ + z^2^) – 1), after which negative values were rounded to zero. This metric is referred to as the Euclidean norm minus one (ENMO) [57]. Raw data were further reduced by calculating the average SVM values per 5-s epoch expressed in *mg* over each of the 16 monitored days.

ActiGraph raw data wear times were estimated on the basis of the standard deviation and value range of each axis, calculated for 60 min moving windows with 15 min increments [57]. A time window was classified as non-wear time if, for at least 2 out of the 3 axes, the standard deviation was less than 13.0 *mg* or if the value range was less than 50 *mg* [57]. A valid day was classified as 10 h or more of accelerometer wear. Participants without 1 valid weekend day each weekend were coded as missing. Moderate-to-vigorous PA (MVPA) derived from the raw accelerations was the primary outcome variable. Wrist-worn specific ActiGraph equations provided by Hildebrand et al. [58] were used to classify MVPA. The Hildebrand equations were solved for 3 METs resulting in MVPA cut-points of 201.4 *mg* and 100.6 *mg* for children and parents, respectively.

#### Physical activity diary

Each participant (i.e., children and parents) was provided with a calendar format paper-based diary to manually record their own PA at the end of each day on each of the 8 weekends. The diary contained separate columns for participants to record the mode (e.g., football, walking) and duration of activity (in minutes), start and end times, location of activity and with whom the activity was undertaken (e.g., on my own, with friend, with brother/sister). Verbal instructions were given to participants by the first author at the first home visit, and an example of a completed entry was provided on the diary to maximise the quality of information provided. Each participant was verbally instructed to record any activity lasting greater than 10 min in duration and were provided with examples of different modes of activity for them to distinguish between unstructured (e.g., outdoor play, active travel) and structured PA (e.g., sport) participation. Diaries were collected from home addresses by the first author after each measurement period. Deductive content analysis was used to explore the diary data [59].

Diary responses were categorised in relation to two higher order themes (e.g., mode of activity and with whom the activity was undertaken), and six lower order themes including unstructured PA (e.g., walking, outdoor play), structured PA (e.g., gym based exercise and activities involving financial cost), club-based/organised PA (e.g., football club and other sporting activities), alone, friend and family (e.g., parent/sibling)) to align with the study objectives. Each recorded entry produced two lower order themes. For example, ‘I played out with friends’ would require marks for unstructured PA and friend. Individual participant responses were summed to produce frequency counts for each lower order theme and then combined to produce an overall frequency count for target children, siblings, mothers and fathers. These were then expressed as a percentage of total number of entries for target children, siblings, mothers and fathers. To ensure accuracy and allow for alternative interpretations of the data, the diaries were independently reviewed by the fourth and final authors and were then cross-examined against the data in reverse, from the frequency counts to the PA diary data sheets. This process was repeated until a 90% agreement level had been reached by the group.

### Statistical analyses

Participant characteristics were analysed descriptively. Variance components in linear mixed models were used to calculate mean MVPA for each weekend, and sources of variability in weekend MVPA for target children (*n* = 7), siblings (*n* = 6), mothers (*n* = 7) and fathers (*n* = 5). Weekend specific MVPA means were calculated by fitting MVPA as the dependent variable, weekend of measurement (1–8) as a fixed effect, and participant (identification number) as a random effect. Weekend of measurement was nested within participants to take the clustering effect of each participant into account. Preliminary analyses confirmed that there were no systematic differences in MVPA or accelerometer wear time due to seasonal/weather variables or accelerometer wear time, therefore these variables were not included as covariates in the variance components models. Variance components were estimated via restricted maximum likelihood estimates using a compound symmetric covariance structure. Variance components were estimated for participant (inter-individual), weekend of measurement, and residual error (intra-individual). Inter-individual variation represents true differences between participants. Weekend variation represents mean differences between weekends. Intra-individual variability represents variation in PA from weekend-to-weekend within participants. The variance components were expressed as a percentage of total variance. To assess the stability of MVPA across weekends, intraclass correlation coefficients were calculated from the proportion of total variance accounted for by inter-individual sources, and used as a measure of reliability (*R*). Analyses were conducted using IBM SPSS Statistics v.23 (SPSS Inc., Chicago, IL, USA). Statistical significance was set at 0.05.

### Family case studies

To provide contextual insight into the characteristics of weekend PA among families, accelerometer, diary, and demographic data for one low active and one high active family were used to produce descriptive case studies. The case study families were purposively selected based on their PA profile from study aim 1. Prior to writing the case studies, the quantitative data were assessed by all authors and consensus was reached that the selected families would allow for the study aims to be achieved. The case studies offer insight into the physical activities that low and high active families undertake on weekend days and demonstrate how this can influence the stability of their weekend PA levels over time. Demographic information in conjunction with accelerometer and PA diary data for contrasting family structures are presented alongside the variance components data (Tables 1 and 2). Pseudonyms were assigned to families and individual case study participants to assure anonymity.

**Table 1:** The Evans Family (Family 1)

The Evans family were of a lower SES than the study average (IMD 36.6 – quantile 5). They live in a terraced house located in an urban residential area. The family comprises a mother and four children (Jamie, aged 10, Mia, aged 8, Liam aged 4 and Izzy aged 2). Miss Evans is healthy weight, unemployed, with high school education. Her MVPA across weekends was low but stable (Fig. 3a) and was amassed through walking and household chores. The Evans children’s weekend PA was completely unstructured in nature. Outdoor play formed the basis of Jamie’s weekend PA. Jamie played outdoors with his friends in the neighbourhood streets and local public green spaces. His MVPA levels were low, and showed no apparent structure or routine across weekends (Fig. 2a). Mia’s weekend physical activities were similar to Jamie’s with the exception that she also often played indoors with her friends and younger siblings. She was more active than Jamie and her MVPA was more consistent than his across weekends. With regards to family-based PA, the Evans family walked a lot on weekend days. However, these bouts of activity varied in duration, ranging from short visits to the local public park to whole-day family outings shopping and visiting the seaside. Subsequently, the Evans children’s MVPA levels, especially Jamie’s were variable across weekends (Fig. 2a and b).

**Table 2:** The Williams family (Family 6)

The Williams family were of a higher SES than the study average (IMD 9.5 - quantile 2). They live in a cul-de-sac located in an affluent suburban neighbourhood with access to a self-contained garden. The family comprises a mother, father, and two children (Olivia, aged 7 and Harry, aged 9). Both parents are healthy weight, degree educated, and in part and full-time employment, respectively. Family-based PA appeared to be a key part of family life. The Williams family amassed their MVPA levels through a combination of organised sport and structured PA. All made regular use of their health club membership. The majority of Mrs. William’s PA took place at the health club and comprised a mixture of gym and group-based exercise. Mr. Williams was also very active (Fig. 3b). On almost all weekends he used the gym at the health club, cycled with friends and coached his local football team. The Williams children recorded high MVPA levels across weekends (Fig. 2a and b). Organised club sport formed the basis of Harry’s and Olivia’s weekend PA. On all but one weekend (weekend 3) Harry played football for his local team and Olivia played Tennis at the health club. The Williams children reported single occurrences of ice skating, swimming, golf, and trampolining, and participated in walking and cycling as a family but on a less regular basis. Despite the Williams children living in a cul-de-sac they reported few experiences of neighbourhood outdoor play. Instead, they used the family garden regularly for active play with friends. Harry’s and Olivia’s PA levels were stable across weekends (Fig. 2a and b) and so were their parents’ (Fig. 3a and b).	

## Results

### Study aim 1

A total of 25 individual participants from 7 families participated. This included 7 ‘target’ children (boys *n* = 4; mean age 10.4 years (SD = 0.6)), 6 other children (siblings; boys *n* = 4, 7.2 years (SD = 0.7)) and 12 adults (mothers *n* = 7; 40.3 years (SD = 5.2); fathers *n* = 5, 41.7 years (SD = 2.8)). Seven weekends were excluded from the analyses for target children and mothers, and 4 weekends were excluded for siblings due to insufficient accelerometer wear time. Therefore, out of a possible 56 weekends, there were 49 weekends of data for target children and mothers. Less data were available for siblings (44 weekends) and fathers (40 weekends). Mean daily accelerometer wear time across weekends was high ranging from 14.2 h (mothers) to 13.4 h (siblings). Descriptive characteristics of the participants are presented in Table 3. With regards to target children, girls were older, heavier and closer to peak height velocity than boys, and had higher BMI, BMI z-scores and IMD scores. Stature and waist circumference were greatest among boys. All target children were classified as healthy weight. With regards to siblings, girls were older, taller and closer to peak height velocity than boys, but boys had higher body mass, BMI and waist circumference than girls. Most siblings were healthy weight (83%). Seventy-one percent of mothers and 60 % of fathers were healthy weight. Mean BMIs for mothers and fathers were 24.5 (SD = 6.3) and 26.5 (SD = 4.8), respectively. Overall mean MVPA was higher among siblings compared to target children, among fathers relative to mothers, and among boys relative to girls for both siblings and target children.

**Table 3:** Characteristics of participants

Variable	Mean ± SD or %	Mean ± SD or %	Mean ± SD or %
	All (*n* = 7)	Boy (*n* = 4)	Girl (*n* = 3)
*Target children*			
Age (years)	10.4 ± 0.6	10.3 ± 0.8	10.6 ± 0.2
Stature (cm)	146.4 ± 5.1	148.6 ± 5.2	143.5 ± 4.0
Mass (kg)	34.8 ± 4.9	34.1 ± 5.6	35.7 ± 4.7
BMI (kg/m^2^)	16.2 ± 1.8	15.4 ± 1.6	17.3 ± 1.7
BMI Z-score	−0.6 ± 1.0	−1.0 ± 1.1	−0.0 ± 0.6
Weight status (%)			
Normal weight	100	100	100
Waist circumference (cm)	63.7 ± 4.7	66.0 ± 4.5	60.6 ± 3.3
Maturity offset (years)	−2.2 ± 1.0	−3.0 ± 0.5	−1.3 ± 0.3
MVPA (mins∙day^−1^)	63.7 ± 33.4	72.5 ± 43.6	52.0 ± 11.85
*Siblings*	All (*n* = 6)	Boy (*n* = 4)	Girl (*n* = 2)
Age (years)	7.2 ± 0.7	7.2 ± 0.7	7.4 ± 1.0
Stature (cm)	127.2 ± 5.4	126.3 ± 6.7	129.0 ± 1.5
Mass (kg)	24.3 ± 5.2	24.8 ± 6.6	23.2 ± 1.8
BMI (kg/m^2^)	14.9 ± 2.1	15.4 ± 2.5	13.9 ± 0.8
BMI Z-score	−0.9 ± 1.7	−0.8 ± 2.1	−1.3 ± 0.6
Weight status (%)			
Normal weight	83.3	75.0	100.0
Overweight	16.7	25.0	0.0
Waist circumference (cm)	59.4 ± 7.7	60.5 ± 9.7	57.2 ± 1.0
Maturity offset (years)	−4.5 ± 0.8	−4.9 ± 0.5	−3.7 ± 0.6
MVPA (mins∙day^−1^)	119.1 ± 41.9	124.6 ± 52.5	108.1 ± 12.7
*Parent*		Male (*n* = 5)	Female (*n* = 7)
Age (years)		41.7 ± 2.8	40.3 ± 5.2
Stature (cm)		179.0 ± 9.8	164.2 ± 3.9
Mass (kg)		84.2 ± 11.4	65.8 ± 16.6
BMI (kg/m^2^)		26.5 ± 4.8	24.5 ± 6.3
Weight status (%)			
Normal weight		60.0	71.4
Overweight		20.0	0.0
Obese		20.0	28.6
MVPA (mins∙day^−1^)		171.5 ± 110.9	130.8 ± 56.2

**Fig. 1:**
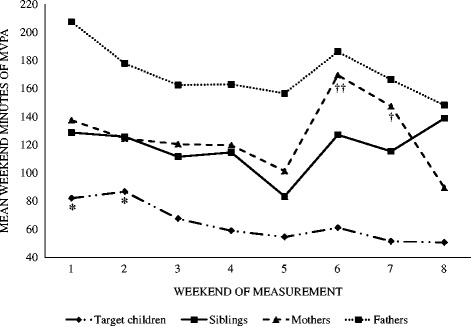
Mean MVPA in target children, siblings, mothers and fathers across measurement weekends

Mean weekend MVPA levels for each weekend of assessment are presented in Fig. 1. There were no significant differences in MVPA between weekends for fathers and siblings respectively. Target children’s and mothers’ MVPA was higher than their mean MVPA level on weekend 1 and 2 (*p* < 0.05), and weekend 6 (*p* < 0.01) and 7 (*p* < 0.05), respectively.

**Fig. 2:**
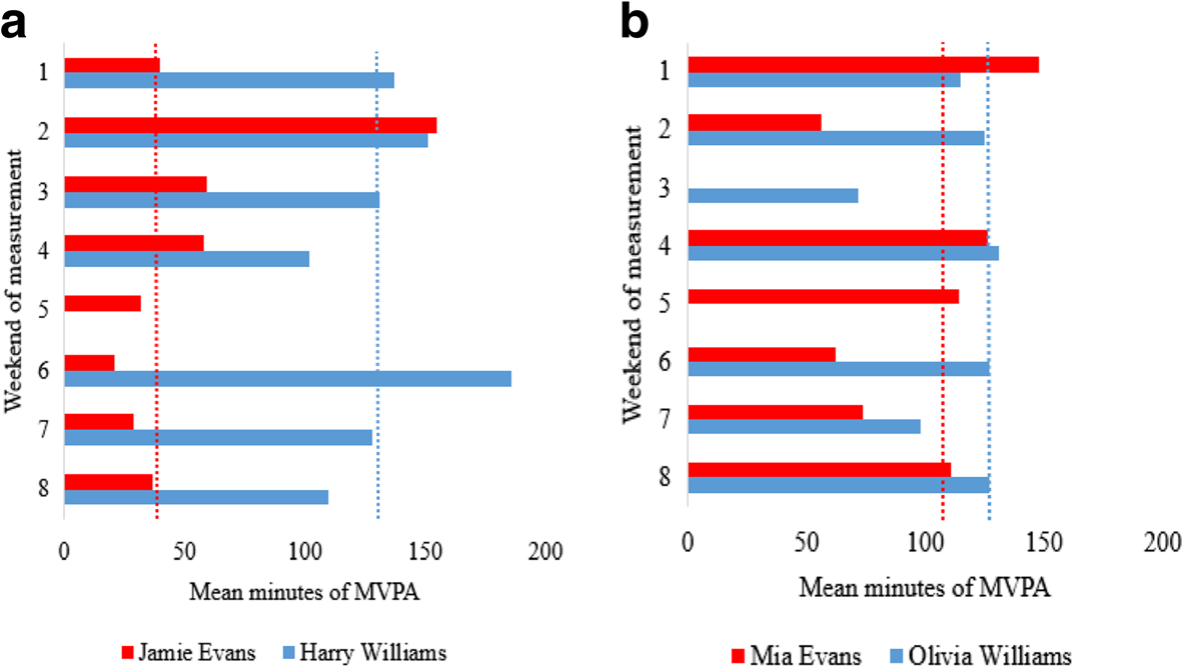
**a**. target children’s mean MVPA comparisons for each weekend. Median MVPA across the 8 weekends for each family is represented by the dotted lines. **b**. siblings’ mean MVPA comparisons for each weekend. Median MVPA across the 8 weekends for each family is represented by the dotted lines

**Fig. 3:**
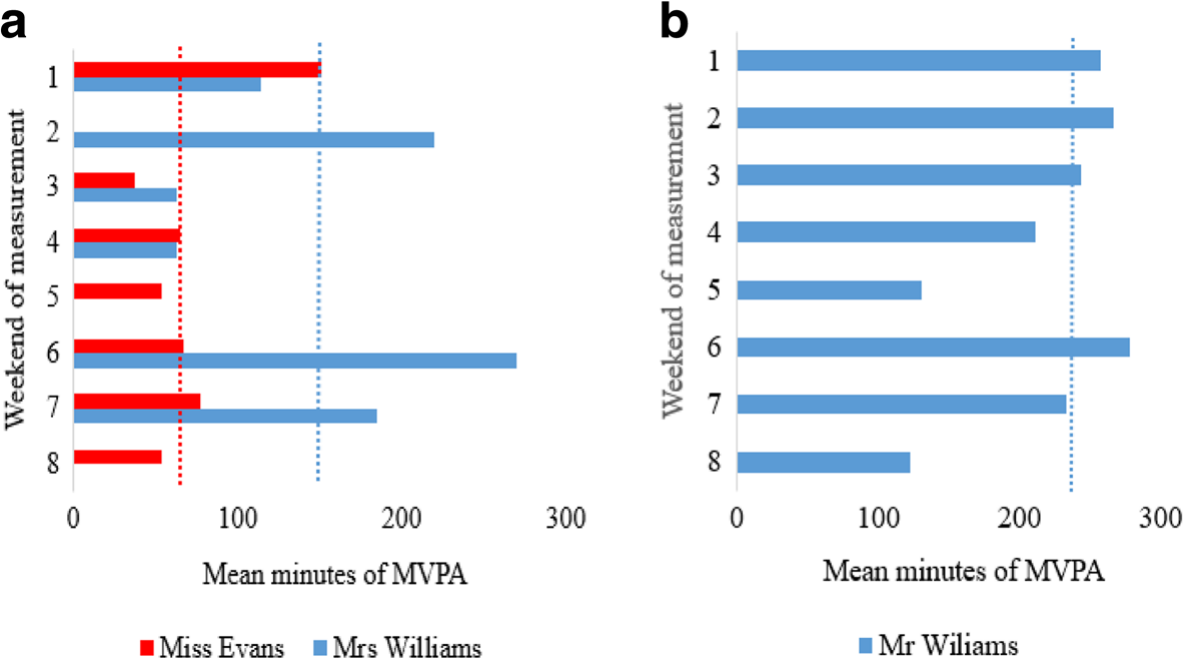
**a**. mothers’ mean MVPA comparisons for each weekend. Median MVPA across the 8 weekends for each family is represented by the dotted lines. **b** fathers’ mean MVPA comparisons for each weekend. Median MVPA across the 8 weekends for each family is represented by the dotted lines

Table 4 displays the sources of variance in MVPA among target children, siblings, mothers and fathers. There was a high degree of variability in target children’s (ICC = 0.55), siblings’ (ICC = 0.38), and mothers’ MVPA across weekends (ICC = 0.58). Fathers’ MVPA was more stable (ICC = 0.83). Total variance was highest in fathers followed by mothers, siblings, and then target children. Inter-individual variance was proportionally the largest source of total variance for target children, siblings, mothers, and fathers but varied considerably (83.1–35.1%). Inter-individual variability was highest in fathers and lowest in siblings. Weekend variance accounted for the second largest source of total variance (9.0–35.0%), followed by intra-individual variability (7.2–27.6%). Intra-individual variance was highest in siblings and lowest in fathers. In models fitted without nesting the weekend effect within participants, the weekend effect was minimal (< 5% of the total variance), and was instead absorbed in the within-participant variance (i.e., residual error). This signified heterogeneous MVPA patterns in the sample for weekend effects.

**Table 4:** Sources of variance in MVPA in target children, siblings, mothers and fathers

	MVPA	
Source of variance	Variance	% of total variance
*Target children*		
Intra-individual	183.7	10.8
Weekend	580.4	34.1
Inter-individual	939.7	55.2
Total	1703.8	
*Siblings*		
Intra-individual	1086.4	27.6
Weekend	1350.7	34.3
Inter-individual	1496.7	38.1
Total	3933.8	
*Mothers*		
Intra-individual	333.7	7.2
Weekend	1631.4	35.0
Inter-individual	2697.7	57.9
Total	4662.8	
*Fathers*		
Intra-individual	1117.2	7.9
Weekend	1279.7	9.0
Inter-individual	11,798.3	83.1
Total	14,195.2	

*MVPA* moderate-to-vigorous physical activity; Percentages may not add to 100% due to rounding

Table 5 presents the PA diary data for target children, siblings, mothers and fathers. There were a combined total of 303 recorded entries for: primary children (*n* = 83), siblings (*n* = 95), mothers (*n* = 73), and fathers (*n* = 52). Target children’s weekend PA time was mostly undertaken with friends (54.2%) and family members (45.8%), and was mainly unstructured in nature (63.9%). Only 4.8% of target children’s weekend PA was undertaken alone. Siblings’ weekend PA was more club-based (41.1%) compared to target children’s (19.3%), and they spent no time alone (0.0%). Mothers’ weekend PA was mostly unstructured (61.6%) and conducted with the family (49.3%) or alone (46.6%). Father’s weekend PA was more structured and club-based (32% and 21.2%, respectively) than mothers (24.7% and 13.7%, respectively) and a greater proportion of fathers’ weekend PA was conducted with friends (11.5%) compared to mothers (4.1%).

**Table 5:** Target children’s, siblings’, mothers’ and fathers’ weekend PA by mode and who they were with

	Mode (%)			Who with (%)		
	Unstructured	Structured	Club/organised	Alone	Friend	Family
Target children (*n* = 83)	63.9	16.9	19.3	4.8	54.2	45.8
Siblings (*n* = 95)	50.5	8.4	41.1	0.0	58.9	41.1
Mothers (*n* = 68)	61.6	24.7	13.7	46.6	4.1	49.3
Fathers (*n* = 52)	46.2	32.7	21.2	38.5	11.5	50.0

*n* = refers to number of entries

### Study aim 2

The descriptive characteristics of families are presented in Table 6. The sample was all white British. The mean IMD score for the sample (26.0 (SD = 11.5)) was slightly higher than the English average (23.6; Public Health England, 2014). Over 50 % of families were degree educated, and all mothers except one had a spouse or partner that was the children’s other parent. All but one family had access to a self-contained garden/backyard. Individual case studies for the Evans and Williams families are presented in Tables 1 and 2, respectively. Mean weekend MVPA levels for the case study families are presented in Figs. 2a (target children), b (siblings), 3a (mothers) and b (father).

**Table 6:** Characteristics of families

Family	IMD (tertile)	Parent education level	Marital status	Target child gender	Sibling gender	Garden/yard
1	36.6 (5)	high school	single, never married	Boy	Girl	No
2	29.5 (4)	university	married	Girl	Boy	Yes
3	42.4 (5)	post-16 college	married	Girl	N/A	Yes
4	19.5 (3)	university	married	Girl	Boy	Yes
5	17.2 (3)	higher degree	married	Boy	Boy	Yes
6	9.5 (2)	university	married	Boy	Girl	Yes
7	27.5 (4)	high school	married	Boy	Boy	Yes

## Discussion

This study used a repeated measures design and multiple data sources to explore the variability and characteristics of weekend PA among families. The study observed substantial variability in children’s weekend PA, and revealed that children’s weekend PA is mostly unstructured in nature and undertaken with friends. The supplementary family case studies (Tables 1 and 2) demonstrated that in the selected cases, MVPA levels and variability across weekends were contingent on mode of PA participation.

The study revealed that parents’ MVPA was more stable across weekends than children’s, and was most stable among fathers (ICC = 0.83) compared to mothers (ICC = 0.58). No previous study has examined PA variability between children and parents, but higher ICC values have been reported in men compared to women for objectively measured total PA over 7 days [60]. A potential reason for the observed difference in PA variability between mothers and fathers in this study may be due to the mode of activity that they undertook. For example, fathers typically engaged in more structured and organised forms of PA (53.8%) compared to mothers (38.4%), and structured PA is known to be more stable relative to unstructured PA [61]. Similar repeated measures studies have been conducted with adults [62, 63]. For example, Levin et al. [61] assessed PA (MET min∙day^−1^) in 77 adults over 48-h every 26 days for 1 year, and reported an ICC value of 0.42. The present study focused on weekend days and comprised a smaller sample and fewer repeated measures compared to the Levin et al. study [62]. These factors are likely to have contributed to the higher ICC estimates observed in the present study.

The ICC values for weekend MVPA in target children (ICC = 0.55) and siblings (ICC = 0.38) in this study are lower than single observation studies in children (ICC = 0.81 [23], ICC = 0.57–0.73 [26], ICC = 0.76–0.97 [28]). However, they are consistent with repeated measures studies [20, 21]. Very few studies have examined variability in children’s weekend PA using accelerometers and a repeated measures design. Mattocks et al. [20] assessed 11- to 12- year-olds’ PA over 7 days on 4 occasions and reported ICC values for total PA (counts per minute) of 0.54 for weekdays and 0.38 for weekend days. Together, these findings demonstrate that a single measurement period is unlikely to accurately represent a child’s typical level of weekend PA, especially among younger children.

The investigation of specific sources of variance in weekend PA revealed that intra-individual variance (i.e., variation in PA from weekend-to-weekend within participants) accounted for a large proportion of total variance among children, especially when models were fitted without nesting the weekend effect within participants. This signified heterogeneous weekend PA patterns. Previous research has shown that children’s PA levels are higher [29, 30] and more stable on weekdays compared to weekend days [20], and most stable during the school day [64]. This is intuitive as the structured school day offers children various formal (e.g., physical education classes, after-school clubs) and informal PA opportunities (e.g., play time/recess) including travelling to and from school actively. When these structures, routines and opportunities are absent on weekend days, children’s PA is more likely to vary from day to day in comparison to weekdays [65]. Moreover, opportunities for PA on weekend days are partly dependent on peer and family-based PA opportunities, strong parental encouragement (e.g., positive verbal reinforcement) and support (e.g., payment of club subscriptions, transport to and from provision) [15, 16, 66], which may also vary from weekend to weekend The combination of these factors may also have contributed to the large intra-individual variability in children’s weekend PA in this study.

The study findings build on previous family-based PA studies [31, 33, 40, 41] by providing contextual insight into weekend PA among family units. Children’s weekend PA was mostly unstructured in nature and undertaken with friends, whereas a greater proportion of parents’ weekend PA was undertaken alone in structured settings. Target children recorded lower MVPA and reported less enrolment in organised/club-based physical activities compared to siblings. This finding supports an age related decline in PA as all the siblings in this study were younger than the target children [67]. However, the finding may also be due to siblings’ more frequent participation in organised sport which is linked with higher child PA [68, 69]. Given that low levels of parent-child co-participation took place in this study future family-based interventions should consider encouraging parents to engage in more physical activity with their children. With regards to family-based PA, popular weekend activities included walking, swimming and visiting public parks. The promotion of these activities may form appropriate intervention settings. Public parks play an important role in supporting PA, providing all families regardless of SES with the opportunity to walk, cycle, and play, with many having specific equipment/activities available for other health enhancing physical activities [70–72]. However, in order to promote regular park use among family units further investment in park programming may be required to provide a variety of features and activities within parks to support the needs of both children and parents [73].

It was apparent from the two family case studies that in the selected cases, the mode of activity families engage in on weekends influences their weekend MVPA levels (Figs. 2 and 3). For example, the Williams’ (i.e., high SES) PA levels were high and structured in nature whereas the Evans’ (i.e., low SES) were low and unstructured in nature. These findings are consistent with previous studies in children [74–76] and adults [77, 78] which reported SES as a strong predictor of PA and organised sport. Weekend leisure opportunities, especially organised ones, generally cost money. Low income families are less likely to have the available logistical and financial resources needed to partake in such leisure opportunities frequently [74, 79, 80]. Therefore, accessible, low-cost weekend PA interventions, such as organised walks, park use or home based activities, may be an appropriate PA intervention for the least active and lowest income families.

The combined use of accelerometers and diaries across multiple weekends provided data that offered contextual insight into the variability of weekend PA among family units. For example, PA levels across weekends were more stable in the Williams family compared to the Evans family (Figs. 2 and 3). The Evans family accrued all of their weekend PA by way of unstructured activities whereas the Williams family participated in activities that were club-based and structured in nature. This finding is intuitive as organised sport participation is linked with higher levels of PA in children [68, 69], and tends to be undertaken regularly, and at predetermined scheduled times. Such structure and routine was evident in Olivia’s and Harry’s PA diary data, but was quite the opposite for Mia and Jamie. By contrast, their PA levels across weekends were more varied, especially Jamie’s (Fig. 2a), and showed no apparent routine or structure. These findings are important as they reveal the potential influence of structured PA participation on habitual weekend PA amongst the selected family units. They suggest that broader intervention approaches such as discounted leisure centre memberships may be needed to provide structured sustainable leisure opportunities for families at weekends [81]. Moreover, as the case study families engaged in few activities together, future child PA interventions may benefit from designing programmes for the whole family.

It is important to understand the barriers to mode-specific weekend PA participation so that strategies can be developed to increase children’s participation in specific modes of weekend PA. The family case studies illustrate the potential environmental barriers to children’s weekend PA and thus highlight the importance of understanding family context and PA characteristics when planning PA interventions. For example, the Williams children have access to a self-contained garden whereas the Evans children do not. This home environmental feature influenced the location of children’s outdoor play. This is a key finding for this family because promoting specific modes of weekend PA (i.e., outdoor play and organised sport) without considering such barriers and constraints is unlikely to support positive sustained behaviour change. As the barriers to participating in organised sport (e.g., financial cost) and unstructured PA (e.g., walkability, access to garden/backyard) are different and vary considerably [17, 82, 83], future PA interventions may be more effective if informed by family characteristics, and tailored to support participation in a specific mode of PA. From a public health perspective, aligning intervention content to the needs, characteristics and constraints of the family will ensure that programmes are relevant and in doing so may positively impact intervention recruitment, engagement and effectiveness.

In addition to these empirical findings, the present study makes a methodological contribution by demonstrating the limitations of one off assessments of weekend PA and single modality PA measurement. The combination of accelerometer and PA diary data allowed exploration of the activities family units undertook on weekend days. By selecting two different family units and comparing their weekend PA behaviours, we were able to demonstrate a way to gain understanding of the complexity of family context, and how, in these cases, family weekend PA varies in mode, location, and variability. Therefore, the findings demonstrate the advantages of supplementing accelerometer data with contextual data, and highlight the importance of distinguishing between structured and unstructured PA participation when examining out-of-school and family-based PA. Future studies in this area may also benefit from the use of ecological momentary assessment (EMA) to obtain ecological real-life data on family PA. EMA collects momentary self-reports in situ, typically implemented as electronic diaries on a handheld electronic device (e.g., smartphone or tablet) [84]. The method would enable the exploration of family weekend PA processes in context, thereby optimising the chance that subsequent intervention programmes based on this knowledge will be effective when employed in daily life [85].

### Strengths and limitations

This is the first study to investigate the variability of weekend PA among children and parents simultaneously. A unique aspect of the study is its repeated measures design. In addition, we used wrist-worn accelerometry and observed high participant compliance to device wear which improves the reliability of PA estimates [86]. Firstly, this provides additional confidence in the study findings, and secondly, offers support that wrist accelerometry is a feasible method of PA assessment in children and adults. Moreover, we assessed weekend PA among families and in doing so revealed new insights into an understudied and complex area of research. The combination of multiple data sources is another strength of the study. Specifically, the combined use of raw accelerometer and diary data allowed exploration of PA mode, location of activity and with whom the activity was undertaken with. However, there are some study limitations. Firstly, our sample size was small, and the participants were all white British and generally healthy weight, which reduced the generalisability of the study. Secondly, participants consented to wearing an accelerometer and completing PA diaries on eight occasions. Therefore, selection factors relating to time availability and study interest may have contributed to a fairly homogeneous sample with active families more inclined to take part. This may have resulted in higher than normal PA levels for the sample. We acknowledge that the case study families are a homogenous group and are unlikely to be those in need of behavioural PA intervention. However, in comparing the two families, it was our aim to demonstrate that weekend PA behaviours differ between families and highlight the need for family-based PA interventions to be tailored to individual needs, characteristics and constraints. Thus, while the findings of this study may not be fully generalisable to other populations and geographical locations, the methods used here are novel and may have wider applicability, and scalability in future health-related research studies involving families.

## Conclusions

The results of this study provide unique information regarding the variability and characteristics of weekend PA among family units. The study demonstrates the potential for using PA diaries in conjunction with accelerometers to provide understanding of the mode and contexts of out-of-school and family-based PA. Future studies using accelerometers should therefore consider the use of PA diaries to provide much needed contextual information. This information can provide contextual understanding as to why some children are more active than others, and may help inform context-specific PA interventions. In addition to promoting family-based weekend PA, strategies to improve neighbourhood design and remove financial barriers to leisure provision are needed. These should be investigated further as components of interventions to promote weekend PA among children and families.

## Abbreviations

BMI: Body mass index
ENMO: Euclidean norm minus one
ICC: Intraclass correlation coefficient
IMD: Indices of multiple deprivation
MVPA: Moderate to vigorous intensity physical activity
PA: Physical activity
SD: Standard deviation
SES: Socioeconomic status
SVM: Signal vector magnitude
